# Abnormal foveal morphology in carriers of oculocutaneous albinism

**DOI:** 10.1136/bjophthalmol-2020-318192

**Published:** 2022-04-04

**Authors:** Helen J Kuht, Mervyn G Thomas, Rebecca J McLean, Viral Sheth, Frank A Proudlock, Irene Gottlob

**Affiliations:** 1 Ulverscroft Eye Unit, Neuroscience, Psychology and Behaviour, University of Leicester, Leicester, UK; 2 Cooper Medical School of Rowan University, Cooper Neurological Institute, Camden, New Jersey, USA

**Keywords:** imaging, macula, retina, vision, genetics

## Abstract

**Background/aims:**

To investigate the foveal morphology in carriers of oculocutaneous albinism (OCA) using spectral domain optical coherence tomography (SD-OCT). A cross-sectional, observational study.

**Methods:**

Handheld SD-OCT (Envisu C2300) was used to acquire horizontal scans through the centre of the fovea in biological parents of patients with OCA (n=28; mean age±SD=40.43±8.07 years) and age-matched and ethnicity-matched controls (n=28; mean age±SD=38.04±10.27 years). Sequence analysis was performed for variants in known genes associated with OCA. Best-corrected visual acuity (BCVA), presence of foveal hypoplasia and grade, foveal, parafoveal and perifoveal thickness measurements of total retinal layers (TRL), inner retinal layers (IRL) and outer retinal layers (ORL) thickness were measured.

**Results:**

Foveal hypoplasia was identified in 32.14% of OCA carriers; grade 1 in all cases. OCA carriers demonstrated significant thicker TRL thickness (median difference: 13.46 µm, p=0.009) and IRL thickness (mean difference: 8.98 µm, p<0.001) at the central fovea compared with controls. BCVA of carriers was between −0.16 and 0.18 logMAR (mean: 0.0 logMAR). No significant differences in BCVA was noted between OCA carriers or controls (p=0.83). In the OCA carriers, we identified previously reported pathogenic variants in *TYR*, *OCA2* and *SLC45A2*, novel *OCA2* variants (n=3) and heterozygosity of the pathogenic *TYR* haplotype.

**Conclusion:**

We have, for the first time, identified foveal abnormalities in OCA carriers. This provides clinical value, particularly in cases where limited phenotype data are available. Our findings raise the possibility that previously reported mild cases of foveal hypoplasia or isolated foveal hypoplasia could correspond to OCA carrier status.

**Key messages**

**What is already known on this topic?**

- Foveal abnormalities have been well described in individuals with oculocutaneous albinism (OCA), with affected individuals demonstrating foveal hypoplasia and an overall increased macular thickness; however, foveal morphology in carriers of OCA has not yet been described.

**What this study adds?**

- We identify subtle foveal abnormalities associated with carriers of autosomal recessive OCA.

**How this study might affect research, practice or policy?**

- Our findings may improve phenotyping in OCA and provide clinical value, particularly in cases where limited phenotype or genotype data are available.

## Introduction

Oculocutaneous albinism (OCA) is a clinically and genetically heterogenous disorder characterised by reduced pigmentation of the hair, skin and the eyes, caused by disruption of the melanin biosynthesis pathway.^1 2^ Ocular characteristics of OCA may include foveal hypoplasia, iris transillumination defect (TID), fundus hypopigmentation, chiasmal misrouting and nystagmus.^2^ Clinical diagnosis of albinism is based on the presence of a combination of these features, or, when genetic diagnosis is confirmed in conjunction with phenotypical characteristics.^2^

OCA is an autosomal recessive disorder, to date mutations of six genes have been associated with non-syndromic forms of OCA.^3^ OCA type 1 is the most common type of albinism in European populations, caused by mutations in the tyrosinase gene (*TYR*).^3 4^ Ocular albinism (OA) is an X linked disorder caused by *GPR143* mutations and characterised by predominantly ocular defects.^5^

Foveal hypoplasia describes the underdevelopment of the fovea, leading to reduced visual acuity. Progressive loss of foveal developmental elements are represented by increasing grades of foveal hypoplasia, as described by Thomas *et al*.^6^ Recent reports show that grades of foveal hypoplasia can predict future vision in preverbal children with nystagmus.^7^ In albinism, higher grades of foveal hypoplasia are often found, however, there have been reports of low grades of foveal hypoplasia or normal foveal development.^2 6^ Ophthalmic characteristics associated with albinism, such as iris TID and fundus hypopigmentation, have also demonstrated significant phenotypic variability.^2^

In autosomal recessive disorders, carriers may present with mild phenotypical characteristics. Carriers of Bardet-Biedl syndrome, neuronal ceroid lipofuscinoses and retinitis pigmentosa have demonstrated abnormal retinal function on electroretinogram testing.^8–10^

Literature reporting ocular characteristics associated with carriers of albinism is scarce. In X linked OA the presence of mud splattered fundus appearance and iris TID in obligate female carriers is reported.^11^ Furthermore, female carriers of *GPR143* (OA) have recently been identified to demonstrate foveal hypoplasia and retinal pigment epithelium mosaicism.^12^

Phenotypical characteristics specifically associated with carriers of autosomal recessive OCA are yet to be reported. The presence of subclinical traits in carriers of other autosomal recessive disorders and OA prompted us to investigate the incidence of ocular abnormalities in OCA carriers, with particular interest in foveal development.

## Methods

### Study population

Recruitment and subsequent investigations occurred at the Leicester Royal Infirmary (LRI), UK, from April 2017 to December 2019.

Sixteen individuals (probands) with OCA were identified at LRI using the clinical diagnostic criteria reported by Kruijt *et al*.^2^ Table 1 shows phenotypical characteristics of probands. Individuals with a diagnosis of OA were excluded.

**Table 1:** An overview of phenotypical characteristics associated with recruited probands, and the foveal morphology description of their biological parents

Probands											Parents	
			Best-corrected VA								Foveal hypoplasia^6 7^	
ID	Gender	Age (at examination)	BE	RE	LE	VA test	Nystagmus	Iris transillumination defect^2^	VEP	Foveal hypoplasia^6 7^	Mother	Father
1	M	5	0.275	0.45	0.275	Crowded Kay Pictures	Present	Grade 1	NT	Grade 1a	Grade 1a	N
2	F	4	0.85	0.85	0.95	Crowded Keeler	Present	Grade 4	Misrouting	Grade 3	N	N
3	F	15	0.18	0.18	0.18	Crowded Keeler	Present	Grade 2	Misrouting	Grade 2	N	NT
4	M	5	0.625	0.75	0.825	Crowded Kay Pictures	Present	Grade 3	Misrouting	Grade 3	Grade 1b	N
5	F	21	0.6	0.78	0.6	Crowded Keeler	Present	Grade 3	Misrouting	Grade 4	N	NT
6	F	22	0.6	0.78	0.6	Crowded Keeler	Present	Grade 2	Misrouting	Grade 3	N	Grade 1a
7	F	13	0.34	0.4	0.4	Crowded Keeler	Present	Grade 2	NT	Grade 4	N	N
8	M	13	0.6	0.64	0.74	Crowded Keeler	Present	Grade 2	Misrouting	Grade 3	N	NT
9	M	22	0.48	0.6	0.48	Crowded Keeler	Present	Grade 3	NT	Grade 3	NT	N
10	M	24	0.48	0.6	0.6	Crowded Keeler	Present	Grade 3	Misrouting	Grade 3	Grade 1a	N
11	M	5	0.9	0.085	0.9	Crowded Kay Pictures	Present	Grade 3	Inconclusive	Grade 3	Grade 1b	N
12	F	10	1.2	1.2	1.28	Crowded Keeler	Present	Grade 3	Misrouting	Grade 4	Grade 1a	N
13	F	4	0.7	0.8	0.75	Crowded Kay Pictures	Present	Grade 4	NT	Grade 3	Grade 1a	N
14	F	3	0.6	NT	NT	Crowded Kay Pictures	Present	Grade 1	Inconclusive	Grade 4	N	Grade 1a
15	M	9	0.78	0.78	0.78	Crowded Keeler	Present	Grade 2	NT	Grade 4	N	N
16	M	3	0.55	0.7	0.575	Crowded Kay Pictures	Present	Grade 2	NT	Grade 3	Grade 1a	N

BE, both eyes; F, female; LE, left eye; M, male; N, normal; NT, not tested; RE, right eye; VA, visual acuity in logMAR; VEP, visual evoked potentia.

Twenty-eight biological parents of confirmed OCA probands were recruited and informed written consent obtained. Exclusion criteria included clinical or genetic diagnosis of albinism, significant ocular and/or neurological disease, or a history of prematurity. All participants were of Caucasian ethnicity.

Twenty-eight, age, refraction and ethnically matched controls were recruited from LRI and the University of Leicester, UK. Exclusion criteria for control participants included history of prematurity, or any significant ocular or neurological disease or history of disease.

### Ophthalmic examination

All carrier and control participants underwent an ophthalmic examination including best-corrected logMAR visual acuity (BCVA) assessment (Precision Vision Visual Acuity Testing V.2.3, Precision Vision, Illinois, USA), detailed orthoptic examination including ocular movements and stereoacuity assessment (Frisby Stereotest).

### Genetic analysis

Saliva samples were collected using Oragene DNA sample collection kits (DNA Genotek, Ontario, Canada) and DNA was extracted as per manufacturer’s guidelines. Genetic analysis was performed using targeted next-generation sequencing, as previously described.^13^ Sequence analysis was performed in 12 probands (75%) (online supplemental table 1). PCR and Sanger sequencing was performed where possible in biological parents. Genotype was obtained in 16 parents (57.1%) (online supplemental table 1). We included the following additional *TYR* variants in our analysis: rs1042602 (c.575C>A: p.(Ser192Tyr)), rs1126809 (c.1205G>A: p.(Arg402Gln)) and rs147546939.^14^

10.1136/bjophthalmol-2020-318192.supp1 Supplementary data

### OCT acquisition

Macular hand-held OCT (HH-OCT) scans were acquired and analysed based on previously approved and published methodology.^15^ High-resolution macular scans were obtained for all participants with a spectral-domain, HH-OCT (Envisu C2300 (Leica Microsystems, Wetzlar, Germany)) mounted on a table with a chin rest, ensuring stability. All HH-OCT examinations of OCA carriers were performed without pupil dilation. The acquisition protocol consisted of a scanning window of 10×5 mm (500 A scans×50 B scans, each B scan averaged 5 times) centred on the macula. Poor quality foveal OCT scans were excluded if the foveal contour was not clearly seen on inspection by expert graders.

Acquired OCT volumetric scans were exported from Leica (Envisu) HH-OCT and analysed using custom-written macros in ImageJ (V.1.48 (National Institutes of Health, Bethesda, Maryland, USA; available at http://rsbweb.nih.gov/ij/; accessed 24 April 2020)). The central scan of the fovea was selected based on the deepest pit. All foveal analysis was performed by examiner(s) masked to the group identity to eliminate bias and all segmentation lines were manually checked for errors.

### Qualitative foveal analysis

The final averaged result was saved as a JPEG file. Five experienced graders inspected all central foveal B scans from carriers and controls. In rare instances, where there were conflicts in grading a senior grader would reassess scans to achieve consensus. Grading of foveal hypoplasia was based on the scheme described by Thomas *et al*, with grade 1 subdivided into 1a and 1b as per Wilk *et al* and Rufai *et al* (online supplemental figure 1).^6 7 16^ Grade 1a foveal hypoplasia was identified by the continuation of the inner retinal layers, the presence of a nearly normal foveal pit, outer segment (OS) layer lengthening and outer nuclear layer widening. A classification of grade 1b foveal hypoplasia was identified where there was continuation of the inner retinal layers, a shallow foveal pit, OS layer lengthening and outer nuclear layer widening. Grade 2 foveal hypoplasia was identified when all features of grade 1 were present, except there was no foveal pit. A classification of grade 3 foveal hypoplasia was identified when all features of grade 2 foveal hypoplasia were present, minus OS lengthening. Grade 4 was identified where all features of grade 3 foveal hypoplasia were present, however there is no outer nuclear layer widening.

10.1136/bjophthalmol-2020-318192.supp2 Supplementary data

### Quantitative foveal analysis

The averaged central B-scans were uploaded to ImageJ to execute quantitative analysis. Retinal layer segmentation was performed using a customised macro in ImageJ, as previously described.^15^ Briefly, this included implementing a spline-based segmentation to derive individual retinal layer thickness.

**Figure 1:**
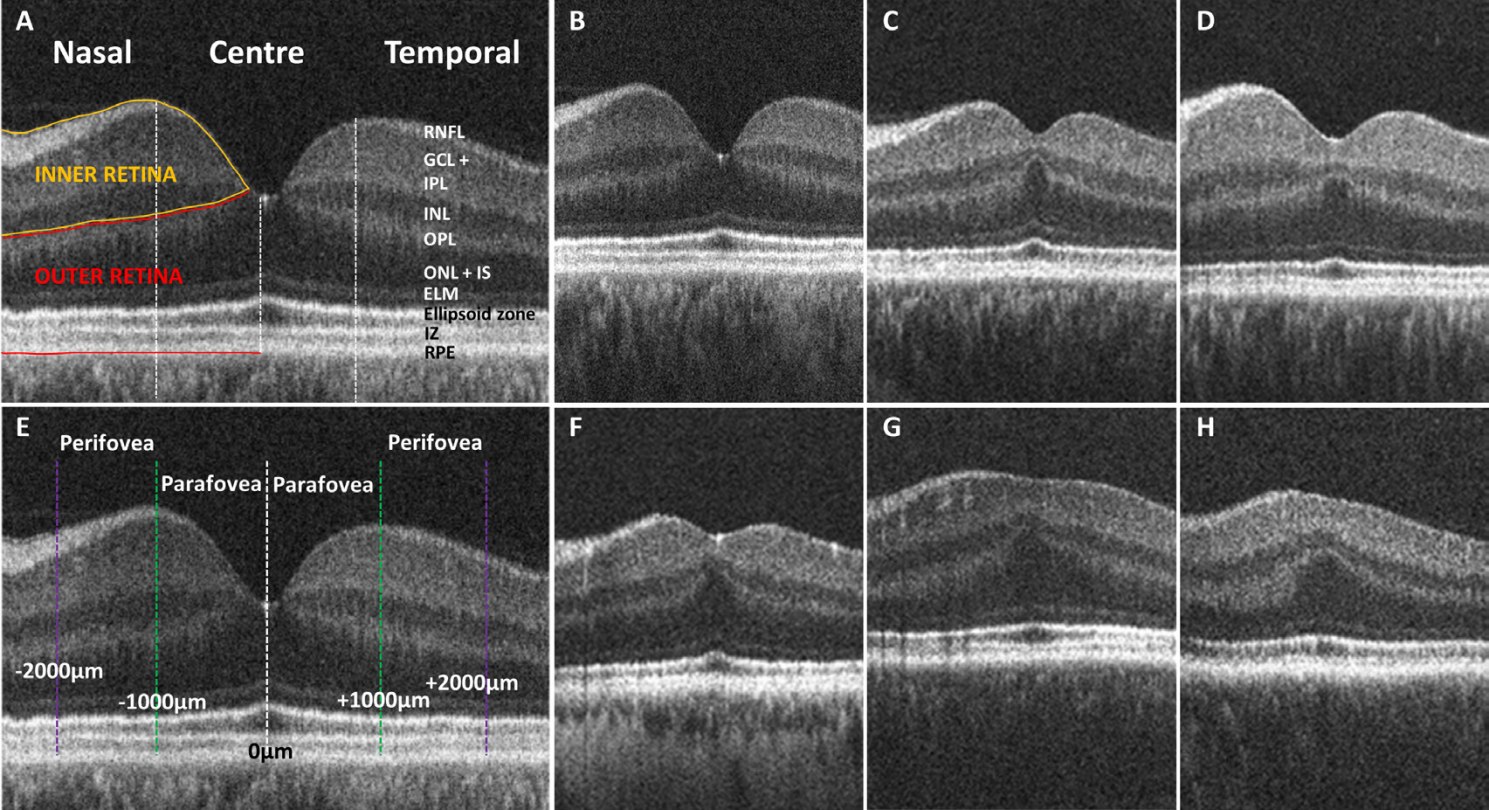
(A) Tomogram representing a horizontal B-scan through the central fovea with indicated retinal layers and central, nasal and temporal measurement locations. Inner retinal layers (RNFL, GCL-IPL complex and INL) are indicated by the yellow border. Outer retinal layers (OPL, ONL, IS, OS and RPE) are indicated by the red border. (E) Thickness measurements were obtained at the central fovea (0 µm; white dashed line) in addition to parafoveal (±1000 µm; green dashed line) and perifoveal locations (±2000 µm; purple dashed line). (B) Foveal tomogram of a control demonstrating normal foveal morphology. (C-D, F) Foveal tomograms of carriers demonstrating grade 1a foveal hypoplasia, with continuation of inner retinal layers posterior to the foveola, a nearly normal foveal pit, OS lengthening and ONL widening. (G–H) Carrier tomograms demonstrating grade 1b foveal hypoplasia with a shallow foveal indent, OS lengthening and ONL widening. ELM, external limiting membrane; GCL, ganglion cell layer; IPL, inner plexiform layer; INL, inner nuclear layer; IS, inner segment; IZ, interdigitation zone; OPL, outer plexiform layer; ONL, outer nuclear layer; RNFL, retinal nerve fibre layer; RPE, retinal pigment epithelium.

Thickness measurements of the total retinal layers (TRL), inner retinal layers (IRL) and outer retinal layers (ORL) were calculated for statistical analysis. IRL comprised retinal nerve fibre layer, ganglion cell-inner plexiform layer complex (GCL-IPL complex) and inner nuclear layer. ORL consisted of outer plexiform layer, outer nuclear layer, external limiting membrane, ellipsoid zone, interdigitation zone and retinal pigment epithelium. Retinal thickness measurements were obtained at 0 µm (central fovea), 1000 µm (parafoveal) and 2000 µm (perifoveal), nasally and temporally from the fovea (figure 1A, E).

### Statistical analysis

Based on retinal thickness measurements from Mohammad *et al*, a sample size of 25 in each group has 80% power to detect a difference between means of 12.16 µm with a significance level (alpha) of 0.05 (two-tailed).^17^ Statistical analysis was executed using SPSS software (V.26.0, IBM, Armonk, New York, USA). Shapiro-Wilk test was applied to test normality of the foveal measurements’ distribution. Retinal thickness measurements at the central fovea demonstrated non-normally distributed data. Retinal thickness measurements at perifoveal and parafoveal regions demonstrated normal distribution.

The Mann-Whitney U test assessed for statistical differences between carriers and controls for retinal thickness at the central fovea for three parameters: TRL, IRL and ORL. For central retinal thickness, the average of right and left eye data was calculated, and subsequently used, for each participant to control for repeated measures. Differences in retinal thickness for each parameter at perifovea and parafovea were compared between the carrier group and the control group using linear mixed models (LMM). LMM were implemented for the analysis of six parameters at both nasal and temporal retinal locations: (1) parafoveal TRL, (2) perifoveal TRL, (3) parafoveal IRL, (4) perifoveal IRL, (5) parafoveal ORL and (6) perifoveal ORL. The independent variable in the LMM was the retinal parameter (ie, parafoveal TRL) and fixed factors included eye (right or left) and group (carrier or control).

All analyses were considered statistically significant when a probability value of p≤0.05 was identified.

## Results

Fifty-six foveal OCT scans from 28 carriers (15 females and 13 males; mean age±SD=40.43±8.07 years) were acquired. Foveal OCT data from one eye of a carrier were excluded from analysis due to a previously identified unilateral localised perifoveal elevation, thus reducing the carrier dataset from 56 eyes to 55. Foveal OCT scans of 55 healthy eyes from 28 controls (17 females and 11 males; mean age±SD=38.04±10.27 years) were subsequently selected for analysis.

### Clinical features

Clinical features for the probands and foveal morphology details of biological parents are described in table 1. All included probands satisfied the clinical/molecular diagnostic criteria for OCA described by Kruijt *et al*.^2^ Carriers had BCVA between −0.16 and 0.18 logMAR (mean±SD=0.00±0.06 logMAR). Controls had BCVA between −0.20 and 0.08 logMAR (mean±SD=−0.02±0.07 logMAR). There was no statistically significant difference between BCVA of carriers and controls (p=0.83) or between the BCVA of carriers with foveal hypoplasia (mean±SD=0.01±0.07 logMAR) and carriers without foveal hypoplasia (mean±SD=−0.02±0.06 logMAR) (p=0.12). However, the two carriers identified with grade 1b foveal hypoplasia demonstrated worse BCVA (0.08 and 0.12 logMAR) than those with no foveal hypoplasia or grade 1a. There was no manifest strabismus for either carriers or controls. All controls and 27/28 carriers (96.4%) demonstrated stereovision of 110” of arc or better. One carrier demonstrated gross stereopsis (600” of arc).

### Genetics

Genetic variants in *TYR, OCA2* and *SLC45A2* were identified. Details of the genetic variants and predicted amino acid changes in probands and parents are shown in online supplemental table 1. Mutation types identified included: missense, deletions and frameshift mutations.

We identified a total of four previously reported pathogenic variants in *TYR*,^18–20^ six variants in *OCA2* 21 22 and one variant in *SLC45A2* (online supplemental table 1).^19^ We reported three novel variants in *OCA2* classed as variants of unknown significance: c.2159G>A: p.(Arg720His); c.2051T>G: p.(Phe684Cys); c.1117–20A>G (online supplemental table 1). The presence of hypomorphic variants in *TYR*: p. Ser192Tyr and p. Arg402Gln are shown in online supplemental table 1.

Overall, among the carriers where sequence analysis was performed, we identified a heterozygous pathogenic variant in 13/16 (81.3%) carriers. In the remaining three carriers (father of proband 1, mother of proband 3 and father of proband 15), interestingly we identified heterozygosity of the recently described *TYR* pathogenic haplotype which is responsible for the partial albinism phenotype in Europeans.^14^

### Visual inspection of foveal morphology

Visual grading of foveal OCT scans identified foveal hypoplasia in 9/28 carriers (32.14%) (figure 1C, D, F–H). All carriers with foveal hypoplasia were recognised as either grade 1a or grade 1b, demonstrating continuation of IRL in conjunction with OS lengthening, widening of the outer nuclear layer and a shallow foveal pit. All controls demonstrated a normal, fully developed foveal pit and no evidence of foveal hypoplasia (figure 1B).

### Foveal measurements

**Figure 2:**
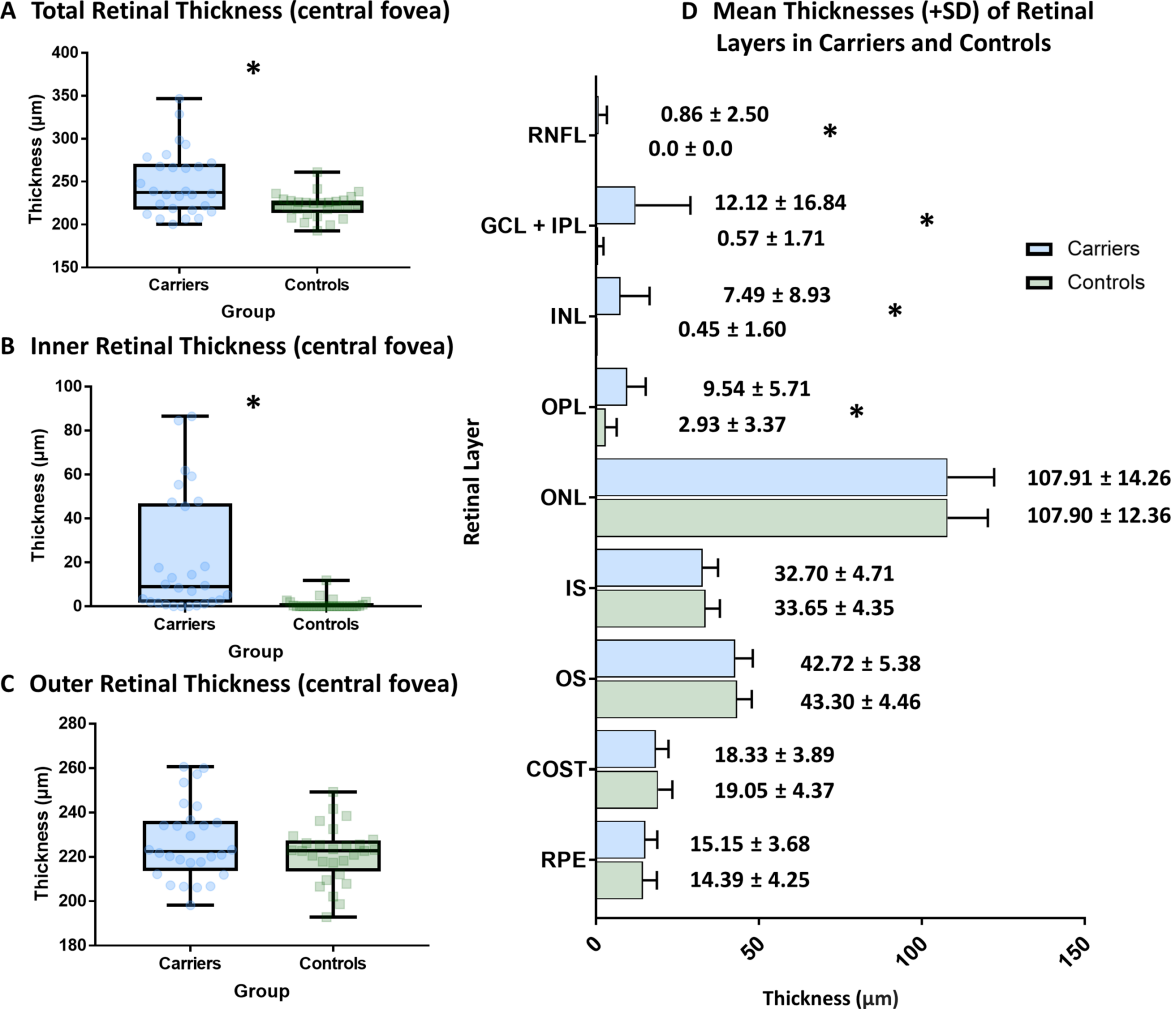
(A–C) Box plot showing the median, IQR and distribution of retinal thickness parameters (TRL, IRL and ORL) in carriers of OCA and healthy controls. (D) Bar graph demonstrating the mean thickness for intraretinal layers at the fovea for OCA carriers and controls. TRL, total retinal layers; IRL, inner retinal layers; ORL, outer retinal layers; OCA, oculocutaneous albinism; RNFL, retinal nerve fibre layer; GCL, ganglion cell layer; IPL, inner plexiform layer; INL, inner nuclear layer; OPL, outer plexiform layer; ONL, outer nuclear layer; IS, inner segment; OS, outer segment; COST, cone outer segment tips; RPE, retinal pigment epithelium; *P<0.05.

Carriers had a significantly thicker TRL (median difference (d): 13.46 µm, p=0.009) (figure 2A) and significantly thicker IRL at the central fovea, compared with controls (d=8.98 µm, p<0.001) (figure 2B). The difference in thickness at central fovea was consistently significant in individual retinal layers: retinal nerve fibre layer, GCL-IPL complex, inner nuclear layer and outer plexiform layer (online supplemental table 2). There was no significant difference between ORL thickness in carriers and controls (d=0.35 µm, p=0.432) (figure 2C).

10.1136/bjophthalmol-2020-318192.supp3 Supplementary data

### Parafoveal and perifoveal retinal layer thickness

**Figure 3:**
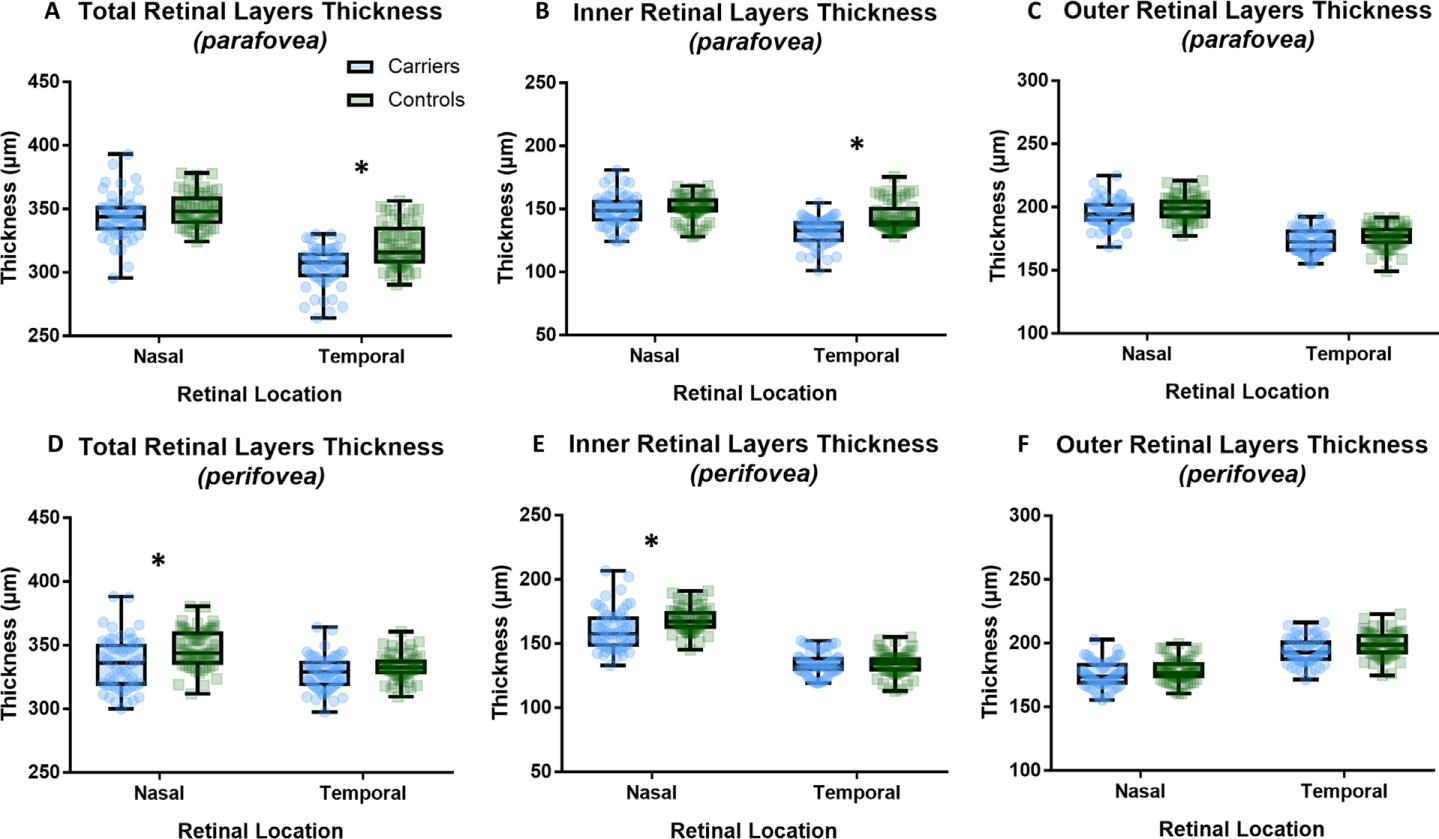
Box plots showing the mean and SD of retinal thickness parameters (total retinal layers, inner retinal layers and outer retinal layers) in the nasal and temporal regions for (A–C) parafoveal retina, and (D–F) perifoveal retina. *P<0.05.

In the temporal parafoveal region, carriers demonstrated a significantly thinner TRL thickness than controls (F=13.89, p<0.001); a result of the significant thinning of the IRL in the temporal parafoveal retina (F=18.51, p<0.001) (figure 3A, B). Temporal IRL thinning occurred due to thinning of the GCL-IPL complex (F=21.71, p<0.001) (online supplemental table 2). We identified significant thinning of perifoveal nasal TRL in carriers (F=5.76, p=0.02) (figure 3D). Perifoveal nasal TRL thinning arises due to significant thinning of the nasal IRL observed in carriers (F=5.72, p=0.02) (figure 3E).

There was no significant effect of groups on parafoveal and perifoveal ORL thickness in either temporal or nasal retina (figure 3C, F, online supplemental table 2). There was no significant effect of eye (right or left) on thickness for all six parameters.

## Discussion

We have described for the first time, foveal abnormalities associated with carriers of autosomal recessive OCA. We identified that approximately a third of our OCA carrier study population (32.1%) had foveal hypoplasia. Of the OCA carriers with foveal hypoplasia, the majority (25%) had grade 1a foveal hypoplasia and only 7.1% had grade 1b. Furthermore, the cohort of all OCA carriers demonstrated an overall increased central macular thickness in comparison to matched controls. Our findings suggest there is an element of arrested retinal development that occurs in OCA carriers, indicative of reduced *TYR* activity despite only carrying one mutant allele.

Despite the presence of foveal hypoplasia, we only found minimal visual consequence in OCA carriers. Visual acuity relies on cone specialisation, which occurs in the ORL. We identified no significant difference of ORL thickness between OCA carriers and controls. Specifically, we find no differences in cone photoreceptor outer segment thickness (figure 2D). Previously, Mohammad *et al* showed that in albinism, visual acuity is correlated to OS length.^17^ Furthermore, Wilk *et al* identified OS length as a surrogate marker for cone spacing, and subsequent visual resolution.^23^ In OCA carriers, we therefore suggest cone photoreceptor specialisation successfully occurs, allowing for high levels of visual acuity to be achieved. Moreover, our identification of only grade 1 foveal hypoplasia in OCA carriers suggests retinal development is disrupted at a later stage of specialisation.

This study has identified significant differences in temporal parafoveal IRL thickness between carriers and controls (figure 3B). In albinism, abnormal projection of retinal ganglion cell axons occurs, where the proportion of temporal retinal fibres projecting onto the contralateral hemisphere is greater than normal.^24^ This midline shift consequently disrupts retinal development and results in a temporal to central shift in albinism. Ganglion cell layer (GCL) thickness in albinism with foveal hypoplasia has previously been identified as demonstrating this shift, consequently resulting in the thinning of the temporal GCL.^25^ Temporal parafoveal IRL thickness in our carrier participants demonstrated significant thinning in comparison to controls, thus indicative of a similar pattern of a temporal to central shift. Interestingly, we recently reported the nasotemporal asymmetry of retinal thickness measurements between patients with *SLC38A8* mutations and controls.^26^ In *SLC38A8* mutations, we find a thinner temporal parafovea with GCL thinning. *SLC38A8* mutations, like albinism have chiasmal misrouting but with no evidence of hypopigmentation. Further studies in other forms of foveal hypoplasia (such as *PAX6* mutations, isolated cases and *AHR* mutations)^27^ will help elucidate whether these observations are specific to the reported temporal to midline shift.

In contrast, the nasal perifoveal IRL thickness was significantly thinner in carriers compared with controls (figure 3E). Ocular maldevelopment in albinism is not only localised to the fovea, but also occurs at the optic nerve head. Peripapillary RNFL thickness has been identified to be significantly thinner in albinism compared with healthy controls, the greatest thinning observed in the temporal quadrant.^28^ Subsequently in albinism, thinning of the temporal peripapillary RNFL extends to the nasal perifoveal RNFL. Our identification of reduced nasal perifoveal IRL thickness in OCA carriers is likely explained by illustrating a similar, although milder, pattern. Further studies investigating optic nerve changes in OCA carriers would provide more insight and enable comparisons with optic nerve changes previously observed in albinism.^28^

For *TYR* and *OCA2* mutations, carrier frequency is reported as approximately 1%.^29 30^ The controversial *TYR* allelic variants resulting in amino acid substitutions p.Ser192Tyr and p.Arg402Gln are frequent in Caucasian populations, with reported minor allele frequencies of 36% and 27%, respectively in European populations.^31^ The exact pathogenicity of these variants are yet to be determined, but there is in vitro evidence of reduced enzymatic activity of tyrosine hydroxylase and DOPA oxidase when the Tyr192 variant allele was compared with the Ser192 wild-type allele.^32^ Similarly, in vitro studies of the R402Q variant have shown the variant results in a thermolabile enzyme that undergoes structural unfolding at higher temperatures with reduced affinity for L-tyrosine resulting in approximately 75% reduction in catalytic activity.^33^

The exact structural and functional consequence of isolated S192Y and R402Q variants are unclear.^3^ The occurrence of continuation of IRL in the general population, with no visual abnormalities, is reported at a frequency of between 1% and 3%.^34^ Thus, raising the question as to whether a potential association between carriers of certain genetic variants and subtle structural abnormalities exists. Furthermore, it is possible that some cases with mild (grade 1) ‘idiopathic foveal hypoplasia’ could be a manifestation of carrier status for albinism, as many people considered with idiopathic foveal hypoplasia are not genetically tested.

Despite the introduction of a clinical classification system for albinism, developed by Kruijt *et al*, many clinicians base albinism diagnosis on the presence of albinotic phenotypical characteristics, irrespective of severity.^2^ Subsequently, there is a large phenotypical spectrum associated with albinism. Previous literature has described cases of partial or mild albinism, which present with low grades of foveal hypoplasia or iris TID, significantly contrasting to the typical presentation of albinism which observes a considerably more severe phenotype.^35^ Our observation of low grades of foveal hypoplasia associated with carriers of OCA may provide one potential explanation for some milder cases of albinism. Previously identified cases of partial albinism may in fact correspond to people with carrier status. Our findings have the potential to improve phenotyping in albinism. Clinical findings of persistence of the IRL in carriers of OA have previously been described.^12^ The observation of foveal abnormalities in carriers of OCA may add clinical value, particularly in cases where limited phenotype data are available (eg, uncooperative infants/young children) and in the absence of a history of prematurity and other ocular disease. Examining and subsequently identifying subtle carrier signs in parents increases the index of suspicion for the child to have albinism and adds to the diagnostic accuracy. The presence of OCA carrier signs may be used as additional criterium for an OCA diagnosis. This study therefore further emphasises the importance of examining the whole family in the context of a suspected Mendelian disorder.

A limitation to our study was the incomplete genetic data, presenting the inability to fully characterise the genotype-phenotype relationships of our carriers. In our study, the number of subjects was relatively small; however, the sample size calculation (see ‘Methods’ section) showed that the power was sufficient.

This adds to the body of evidence demonstrating another autosomal recessive disease in which carriers can be subtly affected. Our findings provide the groundwork for future studies, expanding on the presence of subtle ocular characteristics and the genotype-phenotype relationships in carriers of OCA.

## Data Availability

Data are available on reasonable request. The data generated during and analysed during the current study are available from the corresponding author on reasonable request.
