# Supplementary material for: Abnormal foveal morphology in carriers of oculocutaneous albinism
Source: Br J Ophthalmol. 2022 Apr 4;107(8):1202–8. doi: 10.1136/bjophthalmol-2020-318192 (PMC10359511; doi:10.1136/bjophthalmol-2020-318192)
Supplement: Supplementary data [file bjophthalmol-2020-318192supp001.pdf]

Supplementary table 1 : Genotype of probands and parents.

		Proband			***Mother***			***Father***		
**ID**	**Gender**	**Gene**	**Variant**	**Zygosity**	**Gene**	**Variant**	**Zygosity**	**Gene**	**Variant**	**Zygosity**
1	M	*TYR*	c.575C>A: p.(Ser192Tyr)<br>c.1205G>A: p.(Arg402Gln)<br>c.1217C>T: p.(Pro406Leu)<br>rs147546939 A > G	Heterozygous<br>Heterozygous<br>Heterozygous<br>Heterozygous	*TYR*	c.1217C>T: p.(Pro406Leu)	Heterozygous	*TYR*	c.1205G>A: p.(Arg402Gln)<br>c.575C>A: p.(Ser192Tyr)<br>rs147546939 A > G	Heterozygous<br>Heterozygous<br>Heterozygous
2	F	*SLC45A2*<br>*TYR*	c.593G>A: (p.Gly198Asp)<br>c.575C>A: p.(Ser192Tyr)	Homozygous<br>Homozygous	*SLC45A2*<br>*TYR*	c.593G>A: (p.Gly198Asp)<br>c.575C>A: p.(Ser192Tyr)	Heterozygous<br>Heterozygous	*SLC45A2*<br>*TYR*	c.593G>A: (p.Gly198Asp)<br>c.575C>A: p.(Ser192Tyr)	Heterozygous<br>Heterozygous
3	F	*TYR*	c.238T>C: p.(Trp80Arg)<br>c.575C>A: p.(Ser192Tyr)<br>c.1205G>A: p.(Arg402Gln)<br>rs147546939 A > G	Heterozygous<br>Heterozygous<br>Homozygous<br>Heterozygous	*TYR*	c.575C>A: p.(Ser192Tyr)<br>c.1205G>A: p.(Arg402Gln)<br>rs147546939 A > G	Heterozygous<br>Heterozygous<br>Heterozygous	NP	NP	NP
4	M	NP	NP	NP	NP	NP	NP	NP	NP	NP
5	F	*OCA2*<br>*OCA2*<br>*TYR*	c.1324_1326del:p.(442_442del)<br>c.1117-20A>G[a]<br>c.575C>A: p.(Ser192Tyr)	Heterozygous<br>Heterozygous<br>Heterozygous	*OCA2*	c.1324_1326del:p.442_442del	Heterozygous	NP	NP	NP
6	F	*OCA2*<br>*TYR*	c.1327G>A: (p.Val443Ile)<br>c.575C>A: p.(Ser192Tyr)	Homozygous<br>Homozygous	*OCA2*<br>*TYR*	c.1327G>A: (p.Val443Ile)<br>c.575C>A: p.(Ser192Tyr)	Heterozygous<br>Heterozygous	*OCA2*<br>*TYR*	c.1327G>A: (p.Val443Ile)<br>c.575C>A: p.(Ser192Tyr)	Heterozygous<br>Heterozygous
7	F	*OCA2*	c.1327G>A: (p.Val443Ile)<br>Exon 10 to exon 18 deletion	Heterozygous<br>Heterozygous	NP	NP	NP	NP	NP	NP
8	M	*TYR*	c.74dupT: p.(V25fs)<br>c.575C>A: p.(Ser192Tyr)<br>c.1205G>A: p.(Arg402Gln)<br>rs147546939 A > G	Heterozygous<br>Heterozygous<br>Heterozygous<br>Heterozygous	NP	NP	NP	NP	NP	NP
9	M	*OCA2*<br>*TYR*	c.2159G>A:p.(Arg720His)[a]<br>c.575C>A: p.(Ser192Tyr)<br>c.1205G>A: p.(Arg402Gln)	Heterozygous<br>Heterozygous<br>Heterozygous	NP	NP	NP	NP	NP	NP
10	M	*OCA2*<br>*OCA2*<br>*TYR*	c.1465A>G: (p.Asn489Asp)<br>c.1239+5G>A<br>c.575C>A: p.(Ser192Tyr)	Heterozygous<br>Heterozygous<br>Homozygous	*OCA2*<br>*TYR*	c.1239+5G>A<br>c.575C>A: p.(Ser192Tyr)	Heterozygous<br>Heterozygous	*OCA2*<br>*TYR*	c.1465A>G: (p.Asn489Asp)<br>c.575C>A: p.(Ser192Tyr)	Heterozygous<br>Heterozygous
11	M	*OCA2*<br>*OCA2*<br>*OCA2*<br>*TYR*	c.1327G>A: (p.Val443Ile)<br>c.2051T>G: p.(Phe684Cys)[a]<br>c.2055delT: p.(F685fs)<br>c.575C>A: p.(Ser192Tyr)	Heterozygous<br>Heterozygous<br>Heterozygous<br>Heterozygous	*OCA2*<br>*OCA2*<br>*TYR*	c.2051T>G: p.(Phe684Cys)<br>c.2055delT: p.(F685fs)<br>c.575C>A: p.(Ser192Tyr)	Heterozygous<br>Heterozygous<br>Heterozygous	*OCA2*	c.1327G>A: (p.Val443Ile)	Heterozygous
12	F	NP	NP	NP	NP	NP	NP	NP	NP	NP
13	F	NP	NP	NP	NP	NP	NP	NP	NP	NP
14	F	NP	NP	NP	NP	NP	NP	NP	NP	NP
15	M	*TYR*	c.229C>T: p.(Arg77Trp)<br>c.575C>A: p.(Ser192Tyr)<br>c.1205G>A: p.(Arg402Gln)	Heterozygous<br>Homozygous<br>Heterozygous	*TYR*<br>*TYR*	c.229C>T: p.(Arg77Trp)<br>c.575C>A: p.(Ser192Tyr)	Heterozygous<br>Heterozygous	*TYR*	c.575C>A: p.(Ser192Tyr)<br>c.1205G>A: p.(Arg402Gln)<br>rs147546939 A > G	Heterozygous<br>Heterozygous<br>Heterozygous
16	M	*OCA2*<br>*TYR*	c.1327G>A: (p.Val443Ile)<br>c.1465A>G: (p.Asn489Asp)<br>c.575C>A: p.(Ser192Tyr)	Heterozygous<br>Heterozygous<br>Heterozygous	*OCA2*<br>*TYR*	c.1327G>A: (p.Val443Ile)<br>c.575C>A: p.(Ser192Tyr)	Heterozygous<br>Homozygous	*OCA2*<br>*TYR*	c.1465A>G: (p.Asn489Asp)<br>c.1205G>A: p.(Arg402Gln)	Heterozygous<br>Heterozygous

NP = not performed

[a] These three variants are novel *OCA2* variants.

Accession IDs for transcripts:

TYR = NM_000372

OCA2 = NM_000275

SLC45A2 = NM_016180
