# Supplementary material for: Abnormal foveal morphology in carriers of oculocutaneous albinism
Source: Br J Ophthalmol. 2022 Apr 4;107(8):1202–8. doi: 10.1136/bjophthalmol-2020-318192 (PMC10359511; doi:10.1136/bjophthalmol-2020-318192)
Supplement: Supplementary data [file bjophthalmol-2020-318192supp002.pdf]

Normal foveal structural features detectable using optical coherence tomography	Illustration
(a) Extrusion of plexiform layers<br>(b) Foveal pit<br>(c) OS lengthening<br>(d) ONL widening	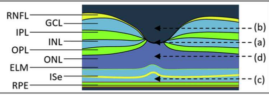

Grade of foveal hypoplasia	Structural features detected on optical coherence tomography	Present or absent	Illustration
**1a**	**(a) Extrusion of plexiform layers**<br>**(b) Foveal pit – Nearly normal**<br>(c) OS lengthening<br>(d) ONL widening	**(a) Absent**<br>(b) Present<br>(c) Present<br>(d) Present	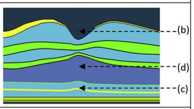
**1b**	**(a) Extrusion of plexiform layers**<br>**(b) Foveal pit – Shallow indent**<br>(c) OS lengthening<br>(d) ONL widening	**(a) Absent**<br>(b) Present<br>(c) Present<br>(d) Present	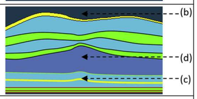
**2**	**(a) Extrusion of plexiform layers**<br>**(b) Foveal pit**<br>(c) OS lengthening<br>(d) ONL widening	**(a) Absent**<br>**(b) Absent**<br>(c) Present<br>(d) Present	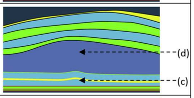
**3**	**(a) Extrusion of plexiform layers**<br>**(b) Foveal pit**<br>**(c) OS lengthening**<br>(d) ONL widening	**(a) Absent**<br>**(b) Absent**<br>**(c) Absent**<br>(d) Present	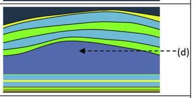
**4**	**(a) Extrusion of plexiform layers**<br>**(b) Foveal pit**<br>**(c) OS lengthening**<br>**(d) ONL widening**	**(a) Absent**<br>**(b) Absent**<br>**(c) Absent**<br>**(d) Absent**	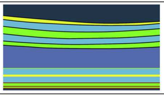
**Atypical**	**(a) Extrusion of plexiform layers**<br>(b) Foveal pit – Shallow<br>(e) ISe disruption	**(a) Absent**<br>(b) Present<br>(e) Present	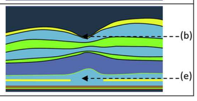

Supplementary figure 1: *Foveal hypoplasia grading scheme by Rufai et al. (2020), adapted from Thomas et al. (2011)*
