# Supplementary material for: Abnormal foveal morphology in carriers of oculocutaneous albinism
Source: Br J Ophthalmol. 2022 Apr 4;107(8):1202–8. doi: 10.1136/bjophthalmol-2020-318192 (PMC10359511; doi:10.1136/bjophthalmol-2020-318192)
Supplement: Supplementary data [file bjophthalmol-2020-318192supp003.pdf]

BMJ Publishing Group Limited (BMJ) disclaims all liability and responsibility arising from any reliance placed on this supplemental material which has been supplied by the author(s)

Supplementary table 2 : Statistical summary of retinal parameters

			Mean ± SD		Linear Mixed Model	
			**Carriers**	**Controls**	**F value**	**P value**
**TRL**	**Nasal**	**Perifoveal**	335.73 ± 21.06	347.31 ± 15.47	5.76	**0.02**
		**Parafoveal**	343.58 ± 17.71	349.20 ± 13.75	1.56	0.215
	**Temporal**	**Parafoveal**	304.56 ± 15.86	320.51 ± 17.46	13.89	**0.000**
		**Perifoveal**	327.98 ± 13.59	333.00 ± 10.45	2.3	0.135
**IRL**	**Nasal**	**Perifoveal**	160.71 ± 16.03	168.86 ± 10.26	5.72	**0.02**
		**Parafoveal**	148.84 ± 12.84	149.76 ± 10.14	0.09	0.769
	**Temporal**	**Parafoveal**	131.79 ± 11.11	143.96 ± 11.46	18.51	**0.000**
		**Perifoveal**	134.32 ± 9.33	134.17 ± 9.54	0.00	0.989
**ORL**	**Nasal**	**Perifoveal**	175.02 ± 10.62	178.45 ± 9.13	1.81	0.184
		**Parafoveal**	194.74 ± 11.51	199.44 ± 9.83	2.73	0.104
	**Temporal**	**Parafoveal**	172.78 ± 9.83	176.54 ± 9.32	2.14	0.149
		**Perifoveal**	193.67 ± 10.48	198.83 ± 10.14	3.34	0.073

	Median		
	**Carriers**	**Controls**	**P Value**
**TRL**	237.5	224.041	**0.009**
**IRL**	8.98	0	**< 0.001**
**ORL**	222.48	222.83	0.432

	Median		
	**Carriers**	**Controls**	**P Value**
**RNFL**	0	0	**0.013**
**GCL +IPL**	3.17	0	**< 0.001**
**INL**	3.03	0	**< 0.001**
**OPL**	9.08	1.66	**< 0.001**
**ONL**	105.53	107.91	0.994
**IS**	32.48	33.5	0.277
**OS**	41.664	43.31	0.546
**COST**	18.44	19.55	0.372
**RPE**	15.43	14.17	0.327

			Mean ± SD		Linear Mixed Model	
			**Carriers**	**Controls**	**F value**	**P value**
**RNFL**	**Nasal**	**Perifoveal**	25.32 ± 4.50	28.67 ± 4.85	13.65	**< 0.001**
		**Parafoveal**	14.68 ± 3.43	13.74 ± 2.76	3.3	0.071
	**Temporal**	**Parafoveal**	11.41 ± 3.08	11.78 ± 2.79	0.48	0.489
		**Perifoveal**	8.77 ± 2.06	9.87 ± 2.25	7.92	**0.006**
**GCL + IPL**	**Nasal**	**Perifoveal**	90.88 ± 11.95	95.26 ± 9.38	4.95	**0.028**
		**Parafoveal**	90.35 ± 10.24	93.00 ± 9.04	1.9	0.172
	**Temporal**	**Parafoveal**	81.04 ± 8.21	88.74 ± 9.39	21.71	**< 0.001**
		**Perifoveal**	83.85 ± 7.58	81.86 ± 7.01	2.08	0.152
**INL**	**Nasal**	**Perifoveal**	44.51 ± 6.85	44.93 ± 5.01	0.14	0.707
		**Parafoveal**	43.80 ± 6.39	43.02 ± 6.12	0.66	0.454
	**Temporal**	**Parafoveal**	39.34 ± 6.81	43.44 ± 4.96	12.99	**< 0.001**
		**Perifoveal**	41.69 ± 6.16	42.44 ± 5.58	0.33	0.565
**OPL**	**Nasal**	**Perifoveal**	29.43 ± 6.5	28.69 ± 4.32	0.43	0.512
		**Parafoveal**	29.50 ± 10.58	28.25 ± 4.94	0.66	0.419
	**Temporal**	**Parafoveal**	24.40 ± 4.41	24.35 ± 4.03	0.01	0.977
		**Perifoveal**	23.82 ± 5.56	23.23.± 4.10	0.49	0.484
**ONL**	**Nasal**	**Perifoveal**	65.33 ± 10.10	69.11 ± 7.41	5.01	**0.027**
		**Parafoveal**	79.89 ± 13.38	84.15 ± 9.03	4.3	**0.041**
	**Temporal**	**Parafoveal**	66.69 ± 6.73	70.04 ± 6.97	7.11	**0.009**
		**Perifoveal**	83.49 ± 6.96	88.07 ± 9.55	9.15	**0.003**
**IS**	**Nasal**	**Perifoveal**	24.77 ± 3.12	26.07 ± 2.75	5.44	**0.022**
		**Parafoveal**	27.93 ± 3.24	29.36 ± 2.90	5.89	**0.017**
	**Temporal**	**Parafoveal**	24.70 ± 2.40	26.32 ± 2.52	11.29	**0.001**
		**Perifoveal**	27.66 ± 2.99	29.05 ± 2.63	6.73	**0.011**
**OS**	**Nasal**	**Perifoveal**	18.32 ± 4.15	18.16 ± 4.62	0.04	0.848
		**Parafoveal**	19.71 ± 3.49	20.50 ± 4.25	1.12	0.293
	**Temporal**	**Parafoveal**	18.76 ± 3.68	18.64 ± 4.88	0.02	0.886
		**Perifoveal**	19.72 ± 3.44	20.66 ± 4.57	1.46	0.230
**COST**	**Nasal**	**Perifoveal**	23.22 ± 3.42	22.37 ± 4.99	1.07	0.302
		**Parafoveal**	23.56 ± 3.58	22.92± 4.46	0.69	0.409
	**Temporal**	**Parafoveal**	22.78 ± 3.90	22.72 ± 4.83	0.01	0.939
		**Perifoveal**	23.63 ± 3.70	22.98 ± 4.76	0.65	0.421
**RPE**	**Nasal**	**Perifoveal**	13.96 ± 3.74	14.04 ± 4.38	0.01	0.922
		**Parafoveal**	14.15 ± 3.83	14.27 ± 4.19	0.01	0.905
	**Temporal**	**Parafoveal**	15.45 ± 3.68	14.47 ± 4.58	1.53	0.219
		**Perifoveal**	15.34 ± 3.45	14.85 ± 4.25	0.46	0.499
